# NK-CD11c+ Cell Crosstalk in Diabetes Enhances IL-6-Mediated Inflammation during *Mycobacterium tuberculosis* Infection

**DOI:** 10.1371/journal.ppat.1005972

**Published:** 2016-10-26

**Authors:** Satyanarayana Swamy Cheekatla, Deepak Tripathi, Sambasivan Venkatasubramanian, Pavan Kumar Nathella, Padmaja Paidipally, Munenori Ishibashi, Elwyn Welch, Amy R. Tvinnereim, Mitsuo Ikebe, Vijaya Lakshmi Valluri, Subash Babu, Hardy Kornfeld, Ramakrishna Vankayalapati

**Affiliations:** 1 Department of Pulmonary Immunology, Center for Biomedical Research, University of Texas Health Science Center at Tyler, Tyler, Texas, United States of America; 2 National Institutes of Health, International Center for Excellence in Research, Chennai, India; 3 Department of Cellular and Molecular Biology, Center for Biomedical Research, University of Texas Health Science Center at Tyler, Tyler, Texas, United States of America; 4 Blue Peter Research Center, LEPRA Society, Cherlapally, Hyderabad, India; 5 Department of Medicine, University of Massachusetts Medical School, Worcester, MA, United States of America; New Jersey Medical School, UNITED STATES

## Abstract

In this study, we developed a mouse model of type 2 diabetes mellitus (T2DM) using streptozotocin and nicotinamide and identified factors that increase susceptibility of T2DM mice to infection by *Mycobacterium tuberculosis* (*Mtb*). All *Mtb*-infected T2DM mice and 40% of uninfected T2DM mice died within 10 months, whereas all control mice survived. In *Mtb*-infected mice, T2DM increased the bacterial burden and pro- and anti-inflammatory cytokine and chemokine production in the lungs relative to those in uninfected T2DM mice and infected control mice. Levels of IL-6 also increased. Anti-IL-6 monoclonal antibody treatment of *Mtb*-infected acute- and chronic-T2DM mice increased survival (to 100%) and reduced pro- and anti-inflammatory cytokine expression. CD11c+ cells were the major source of IL-6 in *Mtb*-infected T2DM mice. Pulmonary natural killer (NK) cells in *Mtb*-infected T2DM mice further increased IL-6 production by autologous CD11c+ cells through their activating receptors. Anti-NK1.1 antibody treatment of *Mtb*-infected acute-T2DM mice increased survival and reduced pro- and anti-inflammatory cytokine expression. Furthermore, IL-6 increased inflammatory cytokine production by T lymphocytes in pulmonary tuberculosis patients with T2DM. Overall, the results suggest that NK-CD11c+ cell interactions increase IL-6 production, which in turn drives the pathological immune response and mortality associated with *Mtb* infection in diabetic mice.

## Author Summary

In the current study, we employed an experimentally induced type 2 diabetes mellitus (T2DM) model in wild type C57BL/6 mice and investigated the immune response to *Mycobacterium tuberculosis* (*Mtb*) infection. We found that natural killer (NK) and CD11c+ cell interactions in *Mtb*-infected T2DM mice led to increased IL-6 production, which drives the pathological immune response and reduces survival of *Mtb-*infected T2DM mice. We also found that IL-6 increases inflammatory cytokine production in pulmonary tuberculosis patients with T2DM. The NK-CD11c+ axis and the IL-6 pathway may be promising new targets for host-directed therapies aimed at reducing the severity of immune pathology, which drives morbidity and mortality in those infected by tuberculosis (TB).The study demonstrates for the first time that NK-CD11c+ cell interactions increase IL-6-mediated inflammation and reduce survival in T2DM mice infected with *Mtb*. The NK-CD11c+ cell axis appears to be a promising new target for reducing inflammation and mortality in tuberculosis patients with type 2 diabetes.

## Introduction

*Mycobacterium tuberculosis* (*Mtb*) infects one-third of the world’s population and causes almost 1.3 million deaths per year [1]. Approximately 90% of those infected have a latent tuberculosis infection and develop protective immunity to contain it; however, but 10% progressive to active tuberculosis (TB) disease months or years after infection [2]. The risk for progression to TB disease is increased by acquired factors including human immunodeficiency virus (HIV) infection, alcoholism, smoking, and diabetes [3].

Developing nations are epicenters of diabetes [4]. Diabetes mellitus alters innate and adaptive immune responses and increases the risk of developing active TB [5]. In type 2 diabetes mellitus (T2DM) patients, there is a reduced association between mycobacteria and monocytes; therefore, phagocytosis via complement receptors is compromised [6,7]. *Mtb*-infected diabetic mice show delayed priming of the adaptive immune response, which is necessary to restrict *Mtb* replication [8]. Hyperactive T-cell responses and increased Th1 and Th17 cytokine production are noted in TB patients with type 2 diabetes [9]. Limited information is available about experimental models used to study the effects of T2DM during *Mtb* infection. Spontaneous T2DM rodent models, such as GK/Jcl rats, have a higher bacterial load and increased immune pathology than non-diabetic Wistar rats after infection with a *Mtb* Kurono aerosol [10]. Furthermore, T2DM guinea pigs are highly susceptible to *Mtb* infection; even non-diabetic hyperglycemia exacerbates disease severity [11,12]. However, a detailed understanding of the protective immune responses in type 2 diabetic hosts during *Mtb* infection is essential if we are to develop an adequate prophylactic or therapeutic agent.

In the current study, we employed an experimentally induced T2DM model in wild type C57BL/6 mice and investigated the immune response to *Mtb* infection. We found that natural killer (NK) and CD11c+ cell interactions in *Mtb*-infected T2DM mice led to increased IL-6 production, which drives the pathological immune response and increases mortality. We also found that IL-6 increases inflammatory cytokine production in pulmonary tuberculosis patients with T2DM.

## Results

### Development of a mouse T2DM model

**Fig 1:**
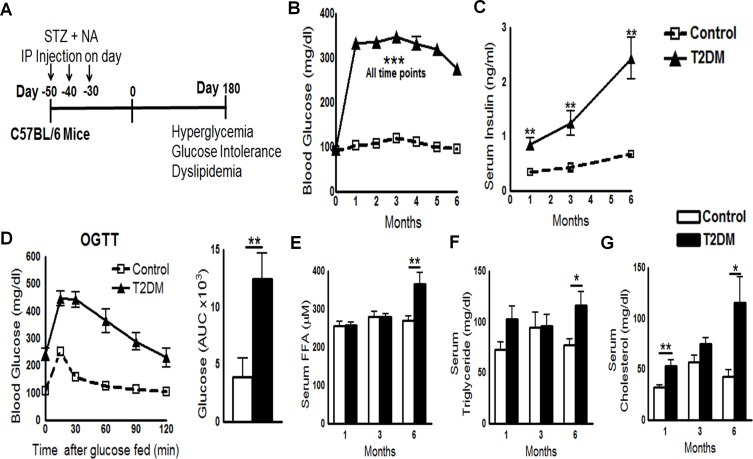
Development of a mouse model of T2DM. **A.** Schematic representation of T2DM induction in C57BL/6 mice. **B.** Random blood glucose sampling at monthly intervals for up to 6 months. Data were pooled from three independent experiments (n = 5 mice per group per experiment). **C.** Serum insulin levels were measured by ELISA at 1, 3, and 6 months. **D.** An oral glucose tolerance test (OGTT) was performed at 6 months post-T2DM induction (left panel). Blood glucose concentrations and the area under the curve are shown (right panel). **E to G.** Serum cholesterol, triglyceride, and free fatty acid levels at 1, 3, and 6 months post-induction of T2DM. **C to G.** Data are representative of two independent experiments (n = 5 mice per group per experiment). *P < 0.05, **P < 0.01, and ***P < 0.001.

A combination of streptozotocin (STZ) and nicotinamide (NA) induces T2DM in mice [8]. STZ (180 mg/kg of body weight) and NA (60 mg/kg of body weight) were administered intraperitoneally to C57BL/6 mice three times, with an interval of 10 days between doses. A schematic representation of T2DM induction is shown in Fig 1A. After 1 month, mice developed T2DM, as assessed by measurement of blood glucose and serum insulin levels. Blood glucose levels measured at monthly intervals for up to 8 months in STZ/NA-treated mice were consistently ≥250 mg/dl (Fig 1B). Blood glucose levels in control mice were 60–125 mg/dl.

To determine whether STZ/NA-treated mice developed insulin resistance, we next measured serum insulin levels. One and three months after T2DM induction, serum insulin levels in STZ/NA-treated mice were significantly higher than those in control mice (Fig 1C). Six months after T2DM induction, serum insulin levels in STZ/NA-treated mice were 4-fold higher than those in control mice (Fig 1C). Insulin resistance, a characteristic feature of T2DM, was confirmed by oral glucose tolerance test (OGTT) 6 months after STZ/NA injection (Fig 1D). Furthermore, serum levels of cholesterol, triglyceride, and free fatty acids were elevated by 6 months after STZ/NA treatment (Fig 1E–1G). Dyslipidemia is another characteristic of T2DM in humans and, combined with demonstrated insulin resistance, confirms the validity of our mouse T2DM model.

### Increased bacterial burden and reduced survival of T2DM mice

**Fig 2:**
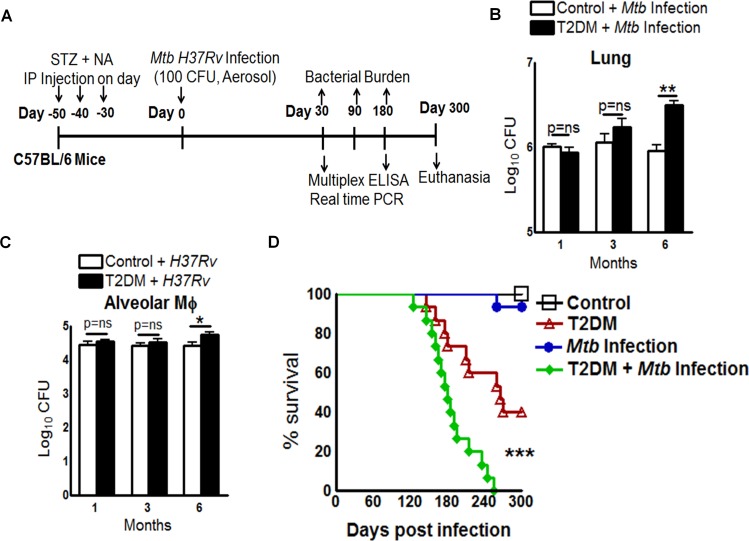
Type 2 diabetes increases the bacterial burden and reduces survival of *Mtb*-infected mice. **A.** Schematic representation of T2DM induction and *Mtb* infection. **B.** Bacterial burden in lungs at 1, 3, and 6 months p.i. Data are representative of two independent experiments (n = 5 mice per group). **C.** Alveolar macrophages from control and T2DM mice (at 1, 3, and 6 months after the induction of diabetes) were infected with *Mtb* at a MOI of 1:2.5. After 2 h, macrophages were washed to remove extracellular bacteria and cultured. After 5 days, intracellular *Mtb* levels were measured. Data points represent the mean value of three independent experiments. Pooled lung alveolar macrophages from two mice per group per time point were used for each independent experiment. **D.** Survival curves for control (black square), T2DM (red triangle), *Mtb*-infected control (blue circle), and *Mtb*-infected T2DM mice (green diamond). Data were pooled from two independent experiments (n = 7–8 mice per group per experiment). Survival curves were compared using the log rank test (P < 0.001). Data are expressed as the mean ± SE. *P < 0.05, **P < 0.01, and ***P < 0.001.

We next investigated TB defense in T2DM mice by aerosol challenge with *Mtb* as shown in Fig 2A. One and three months post-infection (p.i.), the lung bacterial burden was similar in T2DM and control mice (Fig 2B). However, by 6 months p.i., lung bacterial burden was significantly greater in T2DM mice compared to controls (Fig 2B). A similar increase in the bacterial burden was observed in the spleens and livers of T2DM mice when compared with those of control mice (data presented in Dryad Data Repository; doi:10.5061/dryad.qn42t).

Alveolar macrophages are the first immune cells that *Mtb* encounters in the lung [13]. To determine whether the increased bacterial growth described above was due to altered antimicrobial function of these cells, we isolated alveolar macrophages from control and T2DM mice (one, three and six months after T2DM induction) and infected them with *Mtb*. The CFU were quantified after 5 days. *Mtb* growth was similar in the alveolar macrophages of control and T2DM mice after one and three months post induction of T2DM. However, control of *Mtb* growth was impaired in alveolar macrophages, six months after the induction of T2DM (Fig 2C). We next determined the survival of uninfected control and T2DM mice and of *Mtb*-infected control and T2DM mice. By 10 months p.i. all *Mtb*-infected T2DM mice died, whereas only 40% of the uninfected T2DM mice and 6.6% of the *Mtb*-infected non-diabetic mice died (Fig 2D). In contrast, all control mice survived.

### T2DM increases pro- and anti-inflammatory responses during *Mtb* infection

**Fig 3:**
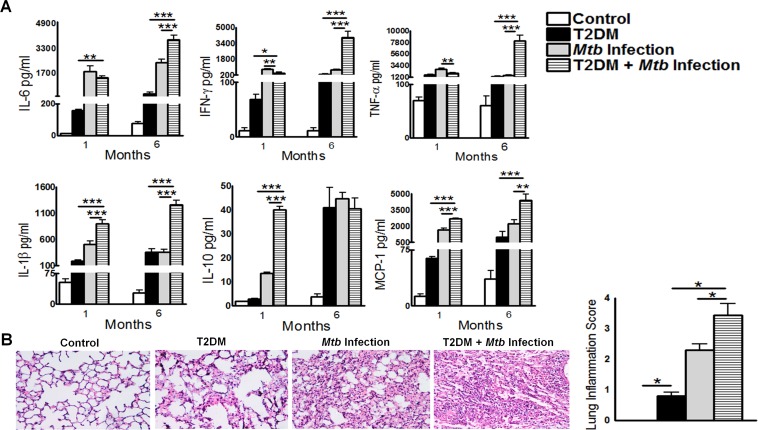
Type 2 diabetes increases pro- and anti-inflammatory responses during *Mtb* infection. Control and T2DM mice were infected with 50–100 CFU of aerosolized *Mtb* H37Rv. **A.** At 1 and 6 months p.i., lung homogenates from uninfected control and T2DM, and *Mtb*-infected control and T2DM, mice were collected and cytokine and chemokine levels were measured in a multiplex ELISA. Data are representative of two independent experiments (n = 5 mice per group per experiment). **B.** At 6 months p.i., lungs from uninfected control and diabetic mice and from *Mtb*-infected control and *Mtb*-infected diabetic mice were isolated and formalin-fixed. Paraffin-embedded tissue sections were prepared, and hematoxylin and eosin staining was performed. Inflamed lung areas were compared between groups. A representative figure is shown. Data are representative of two independent experiments (n = 5 mice per group per experiment). Data are expressed as the mean ± SE. *P < 0.05, **P < 0.01, and ***P < 0.001.

We next determined whether T2DM has any effect on pro- and anti-inflammatory responses following *Mtb* infection. Control and T2DM mice were infected with *Mtb*, and after 1 and 6 months the levels of various cytokines and chemokines were measured in lung homogenates by multiplex (23-plex) ELISA. As shown in Fig 3A, there was a significant increase in both pro- and anti-inflammatory cytokines and chemokines in the *Mtb*-infected T2DM mice at 1 and 6 months p.i., when compared with either uninfected T2DM mice or *Mtb*-infected control mice. However, the cytokine and chemokine levels in the lungs at 6 months p.i. were significantly higher than those at 1 month p.i. The levels of inflammatory cytokines (IL-6, IFN-γ, TNF-α, IL-1β) and chemokines (MCP-1) in whole-lung homogenates from T2DM mice were significantly higher than those in homogenates from *Mtb*-infected control mice or uninfected T2DM mice (Fig 3A). In addition, we found that interleukin (IL)-1α, -5, -9, -12 [p40], -12 [p70], and -13, and G-CSF, GM-CSF, KC, and MIP-1β, in the lung homogenates of *Mtb*-infected T2DM mice were significantly higher than those in *Mtb*-infected control mice and uninfected T2DM mice at 6 months p.i. (data presented in the Dryad Data Repository; doi:10.5061/dryad.qn42t). We also examined expression of various pro- and anti-inflammatory cytokines in whole-lung tissue using real-time PCR. Similar to the ELISA data, we observed increased expression of TNF-α, IFN-γ, IL-6, IL-1β, IL-21, IL-23, TGF-β, and IL-10 in *Mtb*-infected T2DM mice (data presented in the Dryad Data Repository; doi:10.5061/dryad.qn42t).

Histological analysis revealed significantly more inflammation throughout the lungs of *Mtb*-infected T2DM mice when compared with those of *Mtb*-infected control mice or uninfected T2DM mice (Fig 3B).

### IL-6 increases pro- and anti-inflammatory responses and reduces survival of *Mtb*-infected T2DM mice

**Fig 4:**
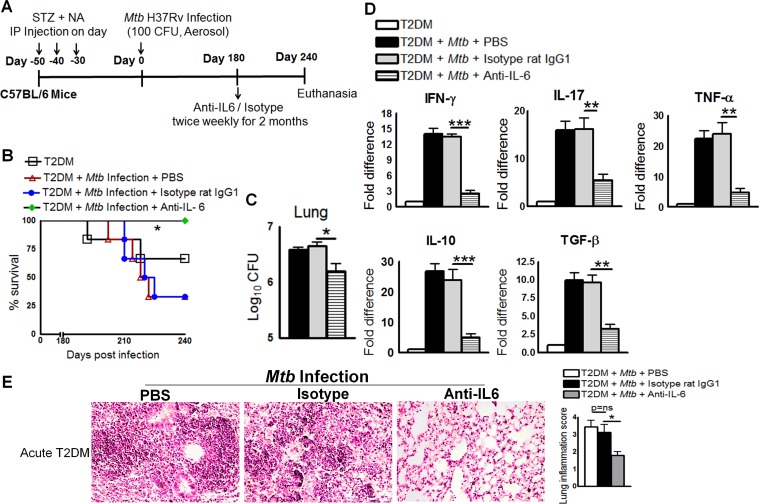
IL-6 increases pro- and anti-inflammatory responses and reduces the survival of *Mtb*-infected mice with acute type 2 diabetes. One month after the induction of diabetes (acute diabetes), T2DM mice were infected with 50–100 CFU of aerosolized *Mtb*. Mice were then treated with either anti-IL-6 mAb or an isotype-matched control mAb (0.3 mg per mouse, starting 6 months p.i. every 4 days for 2 months). **A.** Schematic representation of *Mtb* infection and anti-IL-6 mAb treatment of T2DM mice. **B.** Survival of *Mtb*-infected diabetic mice treated with an anti-IL-6 mAb or an isotype-matched control mAb. Data were pooled from two independent experiments (n = 3 mice per group per experiment). Survival curves were compared using the log rank test (P < 0.001). **C.** Bacterial burden in the lungs. **D.** Cytokine mRNA expression in the lungs, expressed as the -fold difference compared with that in untreated diabetic mice. **C and D.** The lung bacterial burden was determined in four isotype-matched control antibody-treated mice, and RNA was isolated for subsequent mRNA analysis immediately after death. The remaining two isotype-matched control antibody-treated mice and the anti-IL-6 antibody-treated mice were sacrificed at 240 days p.i., to determine the lung bacterial burden and cytokine mRNA levels. T2DM mice and *Mtb*-infected T2DM mice treated with PBS were subjected to the same procedures. **E.** At 6 months p.i., *Mtb*-infected T2DM mice were treated with either an anti-IL-6 mAb or an isotype-matched control mAb or PBS. Lungs were isolated and formalin-fixed. Paraffin-embedded tissue sections were prepared, and hematoxylin and eosin staining was performed. Data were pooled from two independent experiments (n = 3 mice per group per experiment). Data are expressed as mean ± SE. *P ≤ 0.05, **P ≤ 0.01, and ***P ≤ 0.001.

IL-6 is a pleotropic cytokine that regulates both pro- and anti-inflammatory cytokine production [14], and it has both protective and pathogenic roles in diabetes [14]. We found that both pro- and anti-inflammatory cytokine production is dysregulated in *Mtb*-infected T2DM mice compared to control T2DM mice and *Mtb-*infected control mice. There are conflicting reports about the role of IL-6 in *Mtb* infection [15,16]. IL-6-deficient mice are susceptible to *Mtb* infection [15], and IL-6 participates in the induction of type 1 protective T-cell responses after vaccination [17]. However, IL-6 is not required to generate specific immune responses to *Mtb* infection [18]. Thus, we next determined whether neutralizing IL-6 affects survival, cytokine production, or the bacterial burden in T2DM mice. Fig 4A shows a schematic representation of *Mtb* infection and anti-IL-6 mAb treatment in T2DM mice. One month after T2DM induction (acute diabetes), mice were intranasally infected with 50–100 CFU of *Mtb*. At 6 months p.i., the mice were treated with a neutralizing anti-IL-6 mAb, an isotype-matched control mAb, or PBS. As shown in Fig 4B, 65% (p = 0.05) of *Mtb*-infected T2DM (acutely diabetic) mice that received the isotype-matched control mAb or PBS died within 2 months. By contrast, all mice that received the anti-IL-6 mAb survived. Anti-IL-6 mAb treatment also reduced the bacterial burden in the lungs (Fig 4C), spleen (1.5 ± 0.8 × 10^4^ *vs*. 10.5 ± 0.8 × 10^4^ CFU; p = 0.0003), and liver (1.75 ± 0.25 × 10^3^ *vs*. 2.7 ± 0.25 10^3^ CFU; p = 0.03). Real-time PCR analysis of lung samples indicated that anti-IL-6 mAb treatment inhibited expression of IL-17, TNF-α, IL-10, and TGF-β (Fig 4D) when compared with that in mice treated with the isotype-matched control mAb or PBS. Histological examination of lung tissue indicated a similar degree of inflammation in PBS-treated and isotype-matched control antibody-treated mice with acute T2DM and infected with *Mtb* (Fig 4E). By contrast, anti-IL-6 mAb treatment significantly reduced inflammation in the lungs of *Mtb*-infected acute T2DM mice (Fig 4E).

**Fig 5:**
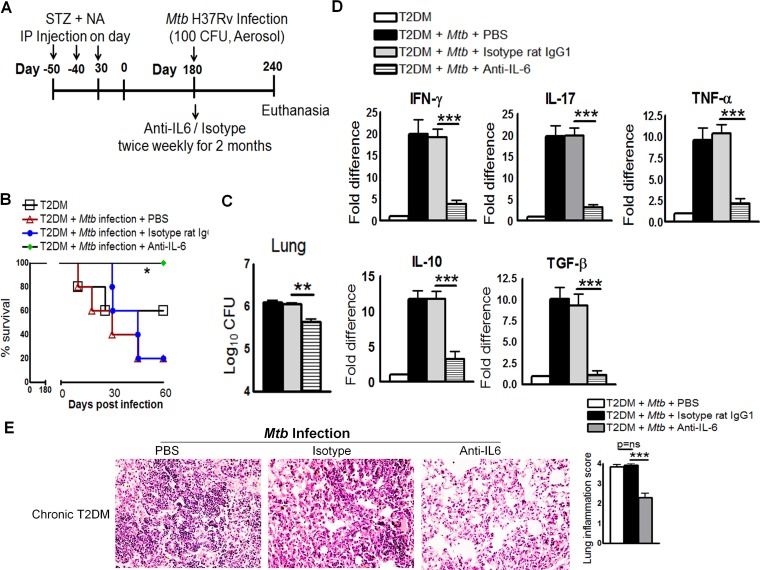
IL-6 increases the pro- and anti-inflammatory responses and reduces the survival of *Mtb*-infected mice with chronic type 2 diabetes. **A.** Six months after the induction of diabetes, T2DM mice were infected with 50–100 CFU of aerosolized *Mtb*. Mice were treated with an anti-IL-6 mAb or an isotype-matched control mAb (0.3 mg per mouse, starting at the time of infection, every 4 days for 2 months). **A.** Schematic representation of *Mtb* infection and anti-IL-6 mAb treatment of T2DM mice **B.** Survival of *Mtb*-infected chronically diabetic mice treated with an anti-IL-6 mAb or an isotype-matched control mAb. Data were pooled from two independent experiments (n = 2 or 3 mice per group per experiment). Survival curves were compared using the log rank test (P < 0.001). **C.** Bacterial burden in the lungs. **D.** Cytokine mRNA expression in the lungs, expressed as the -fold difference compared with that in untreated diabetic mice. **C and D.** The bacterial burden in the lungs of four isotype-matched control antibody-treated mice was determined, and RNA was isolated for mRNA analysis immediately after death. The remaining isotype-matched control antibody-treated mouse and the anti-IL-6 antibody-treated mice were sacrificed at 240 days p.i., to determine the bacterial burden in the lung and cytokine mRNA levels. T2DM mice and *Mtb*-infected T2DM mice treated with PBS were subjected to the same procedures. **E.** Six months after T2DM induction, mice were infected with *Mtb* and treated with either an anti-IL-6 mAb or an isotype-matched control mAb or PBS. Lungs were isolated and formalin-fixed. Paraffin-embedded tissue sections were prepared, and hematoxylin and eosin staining was performed. Data were pooled from two independent experiments (n = 2 or 3 mice per group per experiment). Data are expressed as the mean ± SE. *P ≤ 0.05, **P ≤ 0.01, and ***P ≤ 0.001.

We next determined whether neutralizing IL-6 affected survival, cytokine production, or bacterial burden in mice with chronic T2DM (mice were infected 6 months after T2DM induction). On the day of infection, mice received the anti-IL-6 mAb, the isotype-matched control mAb, or PBS (Fig 5A). As shown in Fig 5B, 80% (p = 0.05) of *Mtb*-infected T2DM mice (chronically diabetic) that received the isotype-matched control mAb or PBS died within 2 months. By contrast, all *Mtb*-infected chronic T2DM mice that received the anti-IL-6 mAb survived. Anti-IL-6 mAb treatment also reduced the bacterial burden in the lungs (Fig 5C), spleen (1.5 ± 0.3 × 10^4^ *vs*. 4.4 ± 0.8 × 10^4^ CFU; p = 0.01), and liver (0.6 ± 0.6 × 10^3^ *vs*. 7.8 ± 1.4 × 10^3^ CFU; p = 0.001). Real-time PCR analysis of lung samples indicated that anti-IL-6 mAb treatment of chronically diabetic *Mtb*-infected mice was associated with reduced expression of IFN-γ, IL-17, TNF-α, IL-10, and TGF-β (Fig 5D) when compared with that in mice treated with the isotype-matched control mAb. Histological examination of the lungs suggested a similar degree of inflammation in PBS-treated and isotype-matched control antibody-treated mice with chronic T2DM harboring *Mtb* (Fig 5E). By contrast, anti-IL-6 mAb treatment significantly reduced inflammation in the lungs of *Mtb*-infected mice with chronic T2DM (Fig 5E).

### CD11c+ cells are the major source of IL-6 in *Mtb*-infected T2DM mice

**Fig 6:**
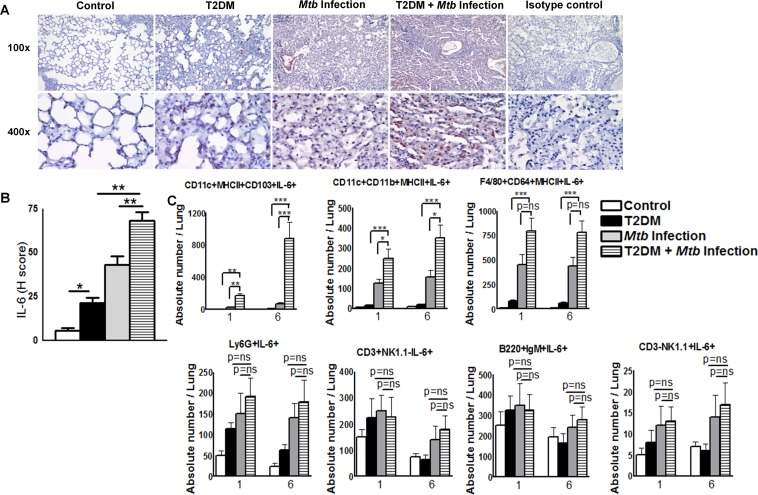
CD11c+CD11b- cells are the major source of IL-6 in *Mtb*-infected type 2 diabetic mice. Control and T2DM mice were infected with 50–100 CFU of aerosolized *Mtb*. **A.** At 6 months p.i., lungs from uninfected control and diabetic mice and from *Mtb*-infected control and diabetic mice were isolated and immunohistochemical analysis of IL-6 expression was performed. Representative images of staining patterns in multiple fields (at 100× and 400×) are shown. **B.** Histological scores for IL-6 expression in lung sections. **C.** The frequencies of IL-6-producing lung cell populations were determined by intracellular flow-cytometry staining at 1 and 6 months p.i. **A to C.** Data are representative of two independent experiments (n = 5 mice per group per experiment). Data are expressed as the mean ± SE. *P ≤ 0.05, **P ≤ 0.01, and ***P ≤ 0.001.

To confirm our finding that IL-6 levels in the lungs of *Mtb-*infected T2DM mice increased at 6 months p.i., mice were euthanized and lung sections were examined for IL-6 expression by immunohistochemistry (IHC). As shown in Fig 6A and 6B, the mean histology score (H-score) for IL-6 in *Mtb*-infected diabetic mice was significantly higher than that in *Mtb*-infected control mice and uninfected diabetic mice. To determine the cellular source of IL-6 in *Mtb-*infected T2DM mice, we first examined the leukocyte populations by flow cytometry. As shown in Table 1, the number of CD11c+MHCII+CD103+, CD11c+CD11b+MHCII+, F4/80+CD64+MHCII+, Ly6G+ neutrophils, and lymphocytes in the lungs of *Mtb*-infected T2DM mice at 1 month post-*Mtb* infection was significantly higher than that in *Mtb*-infected non-diabetic mice or uninfected T2DM. A similar increase was observed at 6 months p.i.

**Table 1:** Leukocyte populations in the lungs of control and *Mtb*-infected mice.

	Months	Control	T2DM	*Mtb* Infection	T2DM + *Mtb* Infection
**CD11c+ MHCII+ CD103+**	**1**	143 ± 66	283 ± 114	868 ± 376	3670 ± 514 * #
	**6**	178 ± 98	211 ± 312	740 ± 253	6660 ± 742 * #
**CD11b+ CD11c+ MHCII+**	**1**	568 ± 262	798 ± 342	3060 ± 797	4740 ± 685 * #
	**6**	778 ± 294	997 ± 385	3170 ±856	5030 ± 768 * #
**F4/80+CD64+ MHCII+**	**1**	2470 ± 562	4200 ± 845	9000 ± 3260	15540 ± 1440 * #
	**6**	1926 ± 429	3940 ± 725	8360 ± 3880	12980 ± 1130 * #
**Ly6G+ Neutrophils**	**1**	2539 ± 751.1	7130 ± 1524	106100 ± 14334	85140 ± 8176 * #
	**6**	2307 ± 652.2	5023 ± 1014	51930 ± 7042	76190 ± 7618 * #
**NKp46+ CD3- NK cells**	**1**	852 ± 68	962 ± 130	3380 ± 288	3330 ± 263
	**6**	585 ± 72	650 ± 113	3074 ± 242	4310 ± 418 * #
**CD3+ NKp46- T cells**	**1**	3120 ± 438	6180 ± 857	15640 ± 2855	27410 ± 2140 * #
	**6**	3790 ± 717	5684 ± 749	11400 ± 2372	14080 ± 3941 * #
**B220+ IgM+ cells**	**1**	14270 ± 1252	21350 ± 2586	35505 ± 1834	49109 ± 1463 * #
	**6**	12600 ± 1418	27135 ± 2586	25501 ± 1995	35767 ± 2228 * #

Control and T2DM mice were infected with 50–100 CFU of aerosolized *Mtb*. At 1 and 6 months p.i., lungs were isolated from uninfected control mice, T2DM mice, *Mtb*-infected mice, and *Mtb*-infected mice with T2DM. The absolute number of leukocytes per 10^6^ total lung cells was determined by flow cytometry. Data are representative of two independent experiments (n = 5 mice per group). Data are expressed as the mean value ± SE.

*p<0.05, T2DM *vs*. T2DM + *Mtb* infection.

**#**p<0.05, *Mtb* infection *vs*. T2DM + *Mtb* infection.

We next examined the phenotype of IL-6 producing pulmonary cells at 1 and 6 months p.i. There were no significant differences in the absolute numbers of IL-6-producing Ly6G+ neutrophils, B220+IgM+ B cells, CD3+NK1.1-T cells, or CD3-NK1.1+ NK cells (Fig 6C). However, the absolute number of IL-6-producing CD11c+MHCII+CD103+ and CD11c+CD11b+MHCII+ cells in the lungs of T2DM mice at 1 month after *Mtb* infection was significantly higher than that in the lungs of uninfected T2DM mice (Fig 6C) or *Mtb*-infected control mice (Fig 6C). As shown in Fig 6C, a similar increase in IL-6-producing CD11c+ cells were noted in the lungs of T2DM mice at 6 months post-*Mtb* infection. Although there was an increased frequency of F4/80+CD64+MHCII+IL-6+ cells in the lungs of *Mtb*-infected T2DM mice compared with those of uninfected T2DM mice at 6 months p.i. (Fig 6C), there was no significant difference between *Mtb*-infected non-diabetic control mice (Fig 6C). Overall, these results suggest that CD11c+ cells are the major source of IL-6 in *Mtb*-infected T2DM mice. To further confirm the cellular source of IL-6 at 6 months p.i., we isolated various cell populations from the pooled spleen, lymph node, and lung cell populations of *Mtb*-infected control and T2DM mice by magnetic sorting and measured IL-6 expression by real-time PCR. We found that CD11c+ cells are the major source of IL-6 (data shown in the Dryad Data Repository; doi:10.5061/dryad.qn42t).

### Interaction between NK and CD11c+ cells increases IL-6 production in *Mtb*-infected T2DM mice

**Fig 7:**
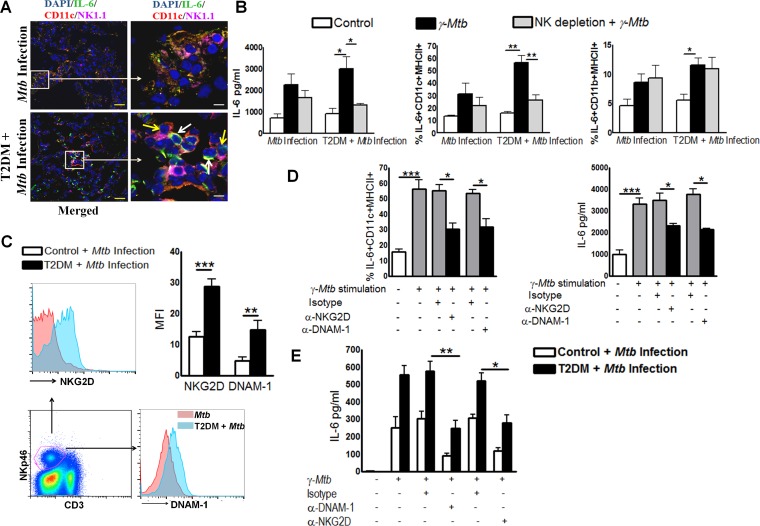
Interaction between natural killer and CD11c+ cells increases IL-6 production in *Mtb*-infected type 2 diabetic mice. T2DM was induced as described in the Methods. Control and T2DM mice were infected with 50–100 CFU of aerosolized *Mtb*. **A.** After 6 months, lungs from *Mtb*-infected T2DM mice were isolated and formalin-fixed. Paraffin-embedded tissue sections were analyzed by confocal microscopy to determine colocalization of NK cells (pink), IL-6+ cells (green), and CD11c+ cells (red). Scale bars, 20 μm (yellow) and 5 μm (white). White arrows point to IL-6-expressing CD11c+ cells, while yellow arrows point to NK1.1+ natural killer cells. A representative staining pattern from three independent experiments is shown (n = 3 mice per group per experiment). **B.** Lung mononuclear cells were isolated by gradient separation and cultured for 48 h with γ-*Mtb* (10 μg/ml). Some mononuclear cell populations were depleted of NK cells and cultured with γ-*Mtb*. The frequency of IL-6-expressing CD11c+ cells was determined by intracellular flow cytometry, and IL-6 levels in culture supernatants were measured by ELISA. **C.** Expression of NKG2D and DNAM-1 by lung NK cells was determined by flow cytometry. **D**. Lung mononuclear cells were isolated as described in the methods section and cultured for 48 h with γ-*Mtb* in the presence or absence of blocking NKG2D or anti-DNAM-1 neutralizing antibodies or an isotype-matched control antibody. The frequency of IL-6+ CD11c+ cells was determined by intracellular staining, and IL-6 levels in culture supernatants were measured by ELISA. **B to D.** Data points represent the mean values from three independent experiments. Pooled lung mononuclear cell populations from two mice per group were used for each independent experiment. **E.** CD3-NK1.1+ and CD11C^+^ cells from pooled spleen, lymph node, and lung cells from *Mtb*-infected control and T2DM mice were isolated by magnetic selection and cultured (one NK cell and four CD11c+ cells) with or without γ-*Mtb* (10 μg/ml). Some of the -irradiated *Mtb* H37Rv-cultured cells were cultured in the presence of blocking antibodies (10 μg/ml) against DNAM-1 or a rat IgG2a κ (the isotype control antibody for the anti-DNAM-1 antibody) or with blocking antibodies against NKG2D or a rat IgG1 κ (the isotype control antibody for the anti-NKG2D antibody). After 18 h, cell-free culture supernatants were collected and IL-6 levels were measured by ELISA. *P ≤0.05, **P ≤ 0.01, and ***P ≤ 0.001.

The interaction between NK cells and macrophages is crucial for the initiation and amplification of early immune responses [19]. We found a significant increase in NK and CD11c+ cell numbers in the lungs of *Mtb*-infected T2DM mice (Table 1). To determine whether NK cells are involved in increased IL-6 production by CD11c+ cells, we first examined lung sections from *Mtb*-infected T2DM mice by confocal microscopy. Imaging results at 6 months p.i. indicated that more NK cells in *Mtb*-infected T2DM mice were in close proximity to IL-6-producing CD11c+ cells than in *Mtb*-infected control mice (Fig 7A and S1 Fig). More importantly, the result indicates that the marked increase in IL-6 production occurs in the region in which both NK cells and CD11c+ cells interact. We further determined whether the NK and CD11c+ cell interaction increases IL-6 production by lung mononuclear cells in *Mtb*-infected T2DM mice. Six months after *Mtb* infection, mononuclear cells were isolated from the lungs of T2DM and non-diabetic control mice and some cell populations were depleted of NK cells by magnetic separation. Lung mononuclear cells and NK cell-depleted lung mononuclear cells were cultured with γ-irradiated *Mtb* H37Rv (γ-*Mtb*). After 48 h, IL-6 levels in the culture supernatants were measured by ELISA and the phenotype of IL-6-producing cells was identified by flow cytometry. Stimulation with γ-*Mtb* significantly enhanced IL-6 production by pulmonary mononuclear cells from *Mtb*-infected T2DM mice (Fig 7B) when compared with those from *Mtb*-infected control mice (Fig 7B). However, depletion of NK cells from *Mtb*-infected T2DM pulmonary mononuclear cells led to a significant reduction in IL-6 levels (Fig 7B). Furthermore, we found that the frequency of IL-6+CD11c+MHCII+ and IL-6+CD11b+MHCII+ cells (Fig 7B) increased significantly after culture of *Mtb*-infected T2DM pulmonary mononuclear cells with γ-*Mtb*. However, depletion of NK cells from *Mtb*-infected T2DM pulmonary mononuclear cells resulted in a significant reduction in the frequency of IL-6+CD11c+MHCII+ cells (Fig 7B). By contrast, depletion of NK cells had no effect on the frequency of IL-6+CD11b+MHCII+ cells (Fig 7B). These results further confirm that CD11c+ cells are a major source of IL-6 and that NK cells from *Mtb-*infected T2DM mice increase IL-6 production by CD11c+MHCII+ cells.

NK cell-activating receptors play an important role in the development of diabetes [20]. We next examined expression of NK cell-activating receptors in *Mtb*-infected mice by flow cytometry. Lung CD3-NKp46+ NK cells from *Mtb*-infected T2DM mice expressed higher levels of NKG2D (Fig 7C) and DNAM-1 (Fig 7C) than those from *Mtb*-infected control mice. We then examined the possible role of these activating receptors in stimulating CD11c+ cells to produce IL-6. At 6 months p.i., lung mononuclear cells from *Mtb*-infected T2DM mice were cultured with γ-*Mtb* in the presence of blocking NKG2D or DNAM-1 mAbs or isotype-matched control antibodies. The frequency of IL-6-expressing CD11c+MHCII+ cells (Fig 7D) increased significantly after culture of *Mtb*-infected T2DM pulmonary mononuclear cells with γ-*Mtb* in the presence or absence of the isotype-matched control antibodies. Blocking the NKG2D (Fig 7D) or DNAM-1 (Fig 7D) interaction with CD11c+ cells led to a significant reduction in the frequency of IL-6+CD11c+ cells. Similarly, IL-6 levels in the culture supernatants of cells cultured with blocking NKG2D (Fig 7D) or DNAM-1 mAbs (Fig 7D) decreased significantly.

To further confirm the above findings, NK cells and CD11c+ cells were isolated from pooled splenic, lymph node, and lung cells from *Mtb*-infected control and T2DM mice by magnetic selection. Autologous NK cells and CD11c+ cells were cultured together at a ratio of 1:4 (1 NK and 4 CD11c+) in the presence or absence of γ-*Mtb* and with or without the isotype control or NKG2D or DNAM-1 blocking antibodies. After 48 h, the culture supernatants were collected and IL-6 levels were measured by ELISA. Culture of *Mtb*-infected control or *Mtb*-infected T2DM mouse NK cells with autologous CD11c+ cells in the absence of γ-*Mtb* did not induce IL-6 production. Culture of *Mtb*-infected control mouse NK cells with autologous CD11c+ cells in the presence of γ-*Mtb* resulted in 251.5 ± 65.1 pg/ml IL-6; this increased to 556.9 ± 52.5 pg/ml (p = 0.02) in NK cells and CD11c+ cells from *Mtb*-infected T2DM mice (Fig 7E). This increase in IL-6 production by CD11c+ cells was inhibited by anti-DNAM-1 and anti-NKG2D blocking antibodies (Fig 7E).

### Anti-NK1.1 antibody reduces pro- and anti-inflammatory responses and increases survival of *Mtb*-infected T2DM mice

**Fig 8:**
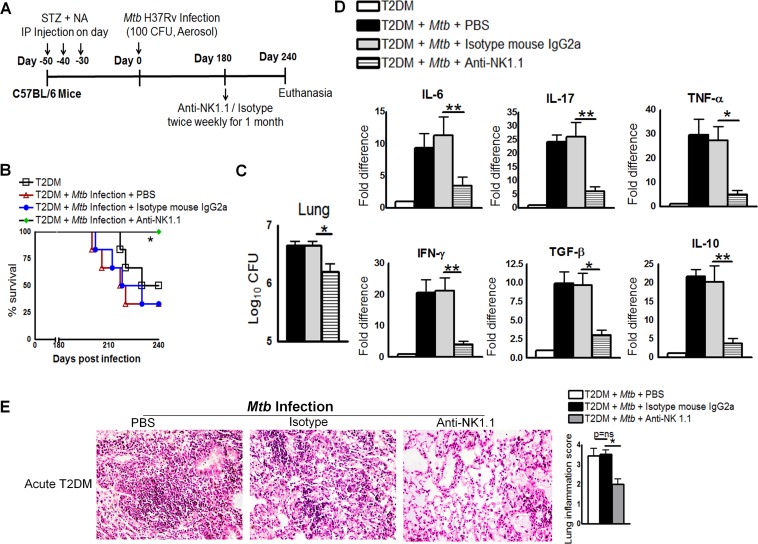
Anti-NK1.1 antibody reduces pro- and anti-inflammatory responses and increases survival of *Mtb*-infected type 2 diabetic mice. One month after STZ/NA treatment, T2DM mice were infected with 50–100 CFU of aerosolized *Mtb* H37Rv. Mice were then treated with an anti-NK1.1 mAb or an isotype-matched control mAb (0.3 mg per mouse, starting at 6 months p.i., every 4 days for 1 month). **A.** Schematic representation of *Mtb* infection and anti-NK1.1 mAb treatment of T2DM mice. **B.** Survival of *Mtb*-infected diabetic mice treated with an anti-NK1.1 mAb or an isotype-matched control mAb. Data were pooled from two independent experiments (n = 3 mice per group per experiment). Survival curves were compared using the log rank test (P < 0.001). **C.** Bacterial burden in the lungs. **D.** Cytokine mRNA expression in the above lung samples, expressed as -fold differences compared with that in untreated diabetic mice. **C and D.** The bacterial burden in the lungs of four isotype-matched control antibody-treated mice was examined, and RNA was isolated for subsequent mRNA analysis immediately after death. The remaining two isotype-matched control antibody-treated mice and anti-NK1.1 antibody-treated mice were sacrificed after 240 days p.i., to determine the bacterial burden in the lung and cytokine mRNA levels. T2DM mice and *Mtb*-infected T2DM mice treated with PBS were subjected to the same procedures. **E.** At 6 months p.i., *Mtb*-infected T2DM mice were treated with either an anti-NK1.1 mAb or an isotype-matched control mAb or PBS. Lungs were isolated and formalin-fixed. Paraffin-embedded tissue sections were prepared, and hematoxylin and eosin staining was performed. Data were pooled from two independent experiments (n = 3 mice per group per experiment). Data are expressed as the mean ± SE. *P ≤ 0.05, **P ≤ 0.01, and ***P ≤ 0.001.

The above result indicated that the interaction between NK and CD11c+ cells increases IL-6 production in *Mtb*-infected T2DM mice. Therefore, we next determined whether depletion of NK cells with a NK1.1 antibody affected survival, cytokine production, or the bacterial burden in acute T2DM mice. One month after STZ/NA treatment, acutely diabetic mice were infected with50–100 CFU *Mtb*. At 6 months p.i., mice were treated with an anti-NK1.1 mAb, an isotype-matched control mAb, or PBS (Fig 8A). As shown in Fig 8B, 65% (p<0.05) of *Mtb-*infected T2DM mice that received the isotype-matched control mAb or PBS died within 2 months. By contrast, all *Mtb*-infected T2DM mice that received the anti-NK1.1 mAb survived. Anti-NK1.1 mAb treatment also reduced the bacterial burden in the lungs (Fig 8C), spleen (1.5 ± 0.86 × 10^4^ *vs*. 10.5 ± 0.86 × 10^4^ CFU; p = 0.0003), and liver (1.75 ± 0.25×10^3^ *vs*. 2.75 ± 0.25 × 10^3^ CFU; p = 0.03) by a marginal, but statistically significant, amount. Real-time PCR analysis of lung samples indicated that anti-NK1.1 mAb treatment of acutely diabetic *Mtb*-infected mice was associated with significantly lower levels of IL-6, TNF-α, IL-10, and TGF-β (Fig 8D) expression than those observed in mice treated with the isotype-matched control mAb. Histological examination of lung tissue indicated a similar degree of inflammation in PBS-treated and isotype-matched control antibody-treated mice with acute T2DM mice and *Mtb* (Fig 8E). By contrast, anti-NK1.1 mAb treatment significantly reduced inflammation in the lungs of *Mtb*-infected mice with acute T2DM (Fig 8E).

### IL-6 increases pro-inflammatory cytokine production in T2DM patients with pulmonary tuberculosis

**Fig 9:**
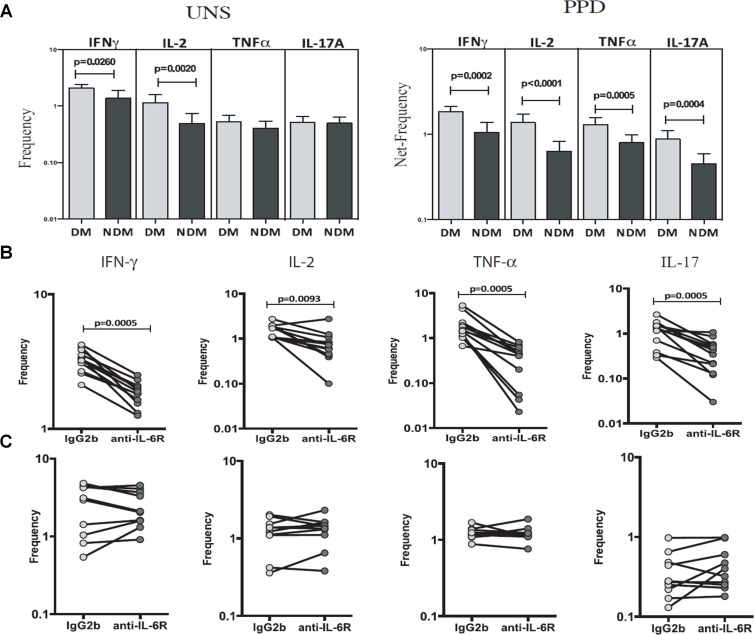
IL-6 drives increased pro-inflammatory cytokine production in type 2 diabetic patients with pulmonary tuberculosis. Blood was obtained from 20 diabetic pulmonary tuberculosis patients (DM) and from 20 non-diabetic pulmonary tuberculosis patients (NDM). Whole blood was stimulated or not (UNS) with 10 μg/ml PPD. **A.** The frequency of IFN-γ-, TNF-α-, IL-17-, and IL-2-producing CD4^+^ T cells was determined by flow cytometry. **B and C.** In some wells, cells from pulmonary tuberculosis patients with T2DM or cells from healthy volunteers were cultured in the presence of anti-IL-6 receptor neutralizing antibodies or isotype-matched control antibodies (2.5 μg/ml). After 18 h, the frequency of IFN-γ-, IL-2-, TNF-α-, and IL-17-producing cells was determined by flow cytometry. **B.** Pulmonary tuberculosis patients with T2DM. **C.** Healthy volunteers. Data are expressed as the mean ± SE. *P ≤ 0.05, **P ≤ 0.01, and ***P ≤ 0.001.

To determine the relevance of the above findings with respect to human *Mtb* infection, we obtained blood from pulmonary tuberculosis patients with or without T2DM and first determined the frequency of pro-inflammatory cytokine-producing T cells by flow cytometry. The frequency of IFN-γ- (1.5-fold, p = 0.026, Fig 9A) and IL-2- (2.1-fold, p = 0.002, Fig 9A) producing cells was significantly higher in the blood of diabetic than in that of non-diabetic pulmonary tuberculosis patients. By contrast, there were no significant differences in the frequency of TNF-α- and IL-17-producing cells between the two groups. We also cultured whole blood in the presence of 10 μg/ml purified protein derivative (PPD). After 18 h, the frequency of IFN-γ-, IL-2-, TNF-α-, and IL-17-producing cells was determined by flow cytometry. As shown in Fig 9A and 9B, PPD significantly induced expression of IFN-γ, IL-2, TNF-α, and IL-17A. The frequency of IFN-γ- (1.7-fold, p = 0.0002; Fig 9A), IL-2- (2.1-fold, p = 0.0001; Fig 9A and 9B), TNF-α- (1.6-fold, p = 0.0005; Fig 9A), and IL-17A- (2-fold, p = 0.0004; Fig 9A) producing cells was significantly higher in diabetic than in non-diabetic pulmonary TB patients. We also examined whether neutralizing the IL-6 receptor affected PPD-induced changes in the frequency of IFN-γ-, IL-2-, TNF-α-, and IL-17A-producing cells in pulmonary TB patients with T2DM. As shown in Fig 9B, the anti-IL-6 antibody significantly reduced the frequency of IFN-γ- (1.7-fold, p = 0.0005), IL-2- (2.2-fold, p = 0.009), TNF-α- (6.6-fold, p = 0.0005), and IL-17A- (3.3-fold, p = 0.0005) producing cells when compared with the isotype-matched control antibody. However, the anti-IL-6 antibody had no effect on the frequency of cytokine-producing cells in healthy volunteers (Fig 9C).

## Discussion

In this study, we investigated the immune response of mice to *Mtb* infection following the induction of T2DM. Diabetic mice were found to have increased lung bacterial burden and mortality compared to non-diabetic controls. Alveolar macrophages from T2DM mice were more permissive to *Mtb* growth ex vivo compared to non-diabetic controls, indicating an impairment of innate antimicrobial function. Multiplex cytokine and chemokine data and real-time PCR analysis of *Mtb-*infected T2DM lungs also demonstrated significantly higher expression of genes encoding pro- and anti-inflammatory cytokines than in lungs from uninfected T2DM and infected non-diabetic control mice. Neutralization of IL-6 increased the survival of all *Mtb*-infected T2DM mice, reduced the bacterial burden, and reduced cytokine production. We found that CD11c+ cells were the major source of IL-6 in *Mtb-*infected T2DM mice. IL-6 production by CD11c+ cells was further enhanced by NK cells. We also found that IL-6 enhances inflammatory cytokine production in pulmonary tuberculosis patients with T2DM. Limited information is available about protective immune responses in type 2 diabetic hosts during *Mtb* infection. Our results suggest that the NK-CD11c+ cell interaction increases IL-6 production, which drives the pathological immune response and reduces survival of *Mtb-*infected T2DM mice.

Chemically induced type 1 diabetes (T1DM) models are widely used in research, and mice with STZ-induced insulin deficiency develop susceptibility to TB [8]. Approximately 90% of people living with diabetes have T2DM, making it by far the most prevalent form of diabetes in TB patients [21]. It is therefore important to employ T2DM models for mechanistic studies of this dual burden. Different approaches have been used to model T2DM in animals, including the combination of high-fat diet with low to intermediate doses of STZ [22]. Some approaches require relatively long periods of time to exhibit all of the major features of the disease and some fail to replicate persistent hyperglycemia [23]. This is an important limitation since the vascular and renal complications of diabetes only develop after prolonged hyperglycemia, and related mechanisms may drive at least some features of diabetic immunopathy [24]. In the current study, we induced T2DM in mice using STZ and NA, which resulted in sustained hyperglycemia for up to 8 months. After 6 months, T2DM mice showed significantly elevated blood cholesterol and triglyceride levels. These findings suggest that our model can be used to investigate TB defenses in the setting of acute or chronic T2DM.

Experimental *Mtb* infection has been investigated in other animal models of T2DM. Sugawara et al. [10] reported that GK/Jcl rats, which spontaneously develop T2DM, have a higher bacterial load and more severe immune pathology than non-diabetic Wistar rats at 5–12 weeks after infection with *Mtb* Kurono. Expression of mRNA encoding several cytokines, including IFN-γ, TNF-β, and IL-1β, was higher in Wistar rats at 1 and 3 weeks p.i., but higher in GK/Jcl rats by 12 weeks p.i. Podell et al. [11] reported increased TB susceptibility in guinea pigs with T2DM induced by low dose STZ plus a high-fat, high-sucrose diet. The guinea pig TB phenotype was characterized by an increased bacterial burden, more severe immune pathology, increased cytokine expression, and increased mortality. The results of TB studies using the rat and guinea pig T2DM models, the mouse T2DM model presented here, and the mouse T1DM model [8] differ in some aspects; however, all show impaired control of *Mtb* replication, more severe immune pathology, and increased expression of multiple cytokines. TB in diabetic people is associated with increased sputum smear positivity at the time of diagnosis (a surrogate marker for higher bacterial burden), increased radiographic severity of disease, increased mortality (reflecting increased immune pathology), and increased expression of several nominally protective cytokines [8,9,25,26]. These similarities support the relevance of our T2DM mouse model to the interaction between TB and diabetes in people.

We found that C57BL/6 mice with STZ/NA-induced T2DM exhibited increased expression of pro- and anti-inflammatory cytokine genes, including IL-6, after *Mtb* infection. IL-6 is a pleiotropic cytokine that has both protective and pathogenic roles in diabetes [14]. There are conflicting reports about the role of IL-6 in *Mtb* infection [15,16]. IL-6 contributes to vaccine-induced protective immunity in mice [17], and IL-6 knockout mice are highly susceptible to *Mtb* [15]. By contrast, IL-6 produced by macrophages infected with *Mtb in vitro* selectively inhibits macrophage responses to IFNγ, thereby contributing to the survival of mycobacteria [27]. Correspondingly, IL-6 neutralization increases IFN-γ-mediated killing of intracellular *Mtb* by inducing autophagy [28]. In human TB patients, IL-6 is implicated in the pathogenesis of the immune reconstitution inflammatory syndrome [29,30]

We found that *in vivo* neutralization of IL-6 conferred a survival benefit and was associated with a reduced bacterial burden in the lung and pro-inflammatory cytokine expression in *Mtb*-infected mice with acute or chronic T2DM; however, it did not alter the hyperglycemic status of the mice. We also found that CD4+ cells from TB patients with T2DM produced significantly elevated levels of Th1 and Th17 cytokines, and that this was inhibited by neutralizing IL-6. A variety of hematopoietic and non-hematopoietic cells produce IL-6, and we found that CD11c+ cells were the major source of IL-6 in *Mtb*-infected T2DM mice. IL-6 production was enhanced by the interaction between NKG2D and DNAM-1 and the corresponding ligands on CD11c+ cells. These results suggest that IL-6 produced during the NK-CD11c+ interaction may be a key factor that drives the damaging immune pathology in diabetic hosts infected by TB. Of note, increased expression of activation markers by unstimulated myeloid cells from diabetic individuals has been documented [31].

NK cells are prominent components of the innate immune system and play a central role in resistance to microbial pathogens. NK cells protect against viruses, parasites, and bacteria, including *Mtb*, by destroying infected cells and secreting cytokines that shape the adaptive immune response [32–34]. NK cells interact with antigen-presenting cells and T cells, and are involved in one or more stages of immune-mediated attack. Abnormalities in the frequency and activity of NK cells have been described both in animal models and in patients with diabetes. By contrast, depletion of NK cells prevents the development of diabetes [35,36]. In TB patients with T2DM, altered CD8+ and NK cell function leads to enhanced pathology [37].

In conclusion, we found that hyperactive NK cells interact with CD11c+ cells to amplify the IL-6-mediated inflammatory immune response in TB. Our data suggest that NK cell-mediated IL-6 production by CD11c+ cells is responsible for driving hyperinflammation and increased mortality in T2DM mice infected with *Mtb*. The mechanism that underlies NK cell hyperactivation in T2DM mice remains unknown, but NK cell activation was recently reported to be an upstream event in T2DM pathogenesis [38]. The NK-CD11c+ axis and the IL-6 pathway may be promising new targets for host-directed therapies aimed at reducing the severity of immune pathology, which drives morbidity and mortality in those infected by TB.

## Methods

### Animals

Specific pathogen-free female wild-type C57BL/6 mice (4 to 6 weeks old) were purchased from Jackson Laboratory and housed at the animal facility at the University of Texas Health Science Center at Tyler. All animal experiments were approved by the Institutional Animal Care and Use Committee of the University of Texas Health Science Center at Tyler.

### Blood donors

Blood was obtained from 20 healthy controls, 20 pulmonary tuberculosis patients, and 20 pulmonary tuberculosis patients with type T2DM. All subjects were HIV-seronegative with culture-proven pulmonary tuberculosis who had received anti-tuberculosis therapy for < 1 week. Acid-fast stains of sputum samples were positive for all patients. Type 2 diabetes patients had HbA1c levels > 6.5% and random blood glucose levels > 200 mg/dl.

### Ethics statement

All human studies were approved by the Institutional Review Board of the National Institute of Research in Tuberculosis (NCT01154959), Chennai, India, and informed written consent was obtained from all participants. All animal studies were approved by the Institutional Animal Care and Use Committee of the University of Texas Health Science Center at Tyler (Protocol #533). All animal procedures involving the care and use of mice were undertaken in accordance with the guidelines of the NIH/OLAW (Office of Laboratory Animal Welfare).

### Antibodies and other reagents

PE-conjugated anti-IL-6 (eBioscience), FITC-conjugated anti-CD3 (Tonbo Biosciences), PE-conjugated anti-NKp46 (BioLegend), PE-cy7 anti-NKG2D (eBioscience), and APC-anti-DNAM-1 (eBioscience) were used for flow cytometry. Antibodies used for the *in vivo* neutralization experiments were purchased from BioXcell (mouse anti-IL-6 [MP5-20F3], anti-NK1.1, and isotype controls [rat IgG1 and mouse IgG2a antibodies]). The NKG2D and DNAM-1 blocking antibodies were obtained from eBioscience. STZ and NA were obtained from Sigma Chemicals. Anti-CD11c, anti-IL-6, anti-NK1.1, secondary antibodies (goat anti-hamster IgG-Alexa 568, donkey anti-rat-Alexa 488, and goat anti-rabbit-Alexa 647), and DAPI were obtained from Life Technologies and used for confocal microscopy. γ-irradiated *Mtb* H37Rv (γ-*Mtb*) was obtained from BEI Resources. Highly purified mouse recombinant IL-6 with a specific activity of 1 10^8^ units/mg was purchased from BioLegend (Bedford, MA).

### Induction of type 2 diabetes

T2DM was induced by combined administration of STZ and NA. STZ was dissolved in a 50 mM citric acid buffer and administered (180 mg/kg of body weight) intraperitoneally three times, with an interval of 10 days between doses. NA was dissolved in saline and administered intraperitoneally (60 mg/kg of body weight) 15 min before STZ. Mice were fasted for 16 h before the STZ and NA injections. Blood glucose was measured using a glucometer at weekly intervals for up to 8 months. Mice were considered diabetic if their blood glucose was > 250 mg/dl. Control mouse blood glucose levels were always between 80 and 100 mg/dl.

### Measurement of serum insulin concentrations

Serum insulin levels in fasting (16 h) control and diabetic mice were measured using a Mercodia Ultrasensitive Insulin ELISA Kit (Mercodia AB Uppsala, Sweden).

### Measurement of serum lipid profiles

Serum free fatty acids, cholesterol, and triglyceride levels were measured using either a fluorometric or colorimetric assay (Cayman Chemicals, USA), according to the manufacturer’s instructions.

### Oral glucose tolerance test (OGTT)

OGTTs were performed in control and diabetic mice after fasting (16 h). A glucose solution (2.0 g/kg) was given orally. Blood glucose concentrations were measured 30 min before and 15, 30, 60, and 120 min after administration.

### Isolation of mouse alveolar macrophages (AMs) and infection with *Mtb* H37Rv

Murine AMs were isolated from control and T2DM mice by bronchoalveolar lavage at 1 and 6 months post-induction of diabetes. Briefly, mice were euthanized by CO_2_ asphyxiation. The trachea was then cannulated following a midline neck incision, and the lungs were lavaged five times with 1.0 ml of ice cold PBS. Alveolar cells were separated from the lavage fluid by centrifugation at 1800 RPM for 10 min. Alveolar cells were plated (on plastic) to permit adherence of alveolar macrophages and subsequent removal of non-adherent NKT and T lymphocytes by washing three times with normal PBS. Adherent cells were resuspended in RPMI-1640. Highly purified AMs were used to determine whether reduced growth of *Mtb* was due to dysfunctional AMs. Alveolar cells were plated in 96-well tissue culture plates at a density of 2 ×10^5^/100 μl/well, incubated for 24 h at 37°C in 5% CO_2_, and washed three times with antibiotic-free RPMI-1640. Around 98% of the cells expressed CD11c, as determined by flow cytometry. AMs were infected with *Mtb* H37Rv at a MOI of 1:2.5 (2.5 *Mtb* to 1 macrophage). This MOI was based on the viability of AMs at different MOIs for up to 7 days p.i. More than 90% of AMs were viable at this MOI. Cells were incubated for 2 h at 37°C in a humidified 5% CO_2_ atmosphere, washed to remove extracellular bacilli, and cultured in RPMI 1640 containing 10% heat-inactivated human serum. To quantify the intracellular growth of *Mtb* H37Rv, infected AMs were cultured for 5 days after which the supernatant was aspirated and AMs were lysed. Bacterial suspensions in cell lysates were ultrasonically dispersed, serially diluted, and plated in triplicate on 7H10 agar. The number of colonies was counted after 3 weeks.

### Aerosol infection of mice with *Mtb* H37Rv

Before infecting mice with *Mtb* H37Rv, bacteria were grown in liquid medium to the mid-log phase and then frozen in aliquots at -70°C. Bacterial counts were determined by plating on 7H10 agar supplemented with oleic albumin dextrose catalase (OADC). For infection, bacterial stocks were diluted in 10 ml of normal saline (to 0.5 ×10^6^ CFU [colony forming units]/ml, 1 ×10^6^ CFU/ml, 2 ×10^6^ CFU/ml, and 4 × 10^6^ CFU/ml) and placed in a nebulizer within an aerosol exposure chamber custom made by the University of Wisconsin. In preliminary studies, groups of three mice were exposed to the aerosol at each concentration for 15 min. After 24 h, mice were euthanized and homogenized lungs were plated on 7H10 agar plates supplemented with OADC. CFUs were counted after 14–22 days of incubation at 37°C. The concentration that deposited ~75–100 bacteria in the lung during aerosol infection was used for further studies.

### Neutralization of IL-6

For some experiments, mice were treated with neutralizing anti-IL-6 antibodies. For the first set of experiments, conducted 1 month after the induction of T2DM, mice were challenged with aerosolized *Mtb*. At 6 months p.i., mice received 0.3 mg of anti-IL-6 mAb (BioXcell) or isotype-matched control Ab (rat IgG1) intravenously every 4 days for up to 2 months. For the next set of experiments, conducted at 6 months after the induction of T2DM, mice were infected with *Mtb* H37Rv. On day “0” of infection, mice received 0.3 mg of anti-IL-6 or isotype control Ab intravenously every 4 days for up to 2 months.

### Depletion of NK1.1+ cells

One month after the induction of T2DM, mice were infected with aerosolized *Mtb*. On day “0” of infection, mice received 0.3 mg of anti-NK1.1 mAb or isotype control Ab intravenously every 4 days for up to 1 month. Previously, we used the same anti-NK1.1 (PK136) antibody to deplete 95% of the NK1.1 cells [34].

### Isolation and culture of lung mononuclear cells

Lungs from *Mtb*-infected mice were mechanically homogenized and filtered through a 70 μm cell strainer. Cells were washed twice, and mononuclear cells were isolated from lung single-cell suspensions using a one-step gradient separation method (GE Healthcare), according to the manufacturer’s instructions. Some mononuclear cell populations were depleted of NK cells by positive selection using antibody-labeled magnetic beads (Miltenyi Biotech), according to the manufacturer’s instructions. Lung mononuclear cells and NK cell-depleted mononuclear cells were seeded in 12-well culture plates (1 × 10^6^ cells per well) and cultured with γ-*Mtb*. After 48 h, cells were harvested and IL-6 expression was measured by intracellular flow cytometry. Supernatants were also collected to measure IL-6 levels by ELISA.

### Isolation of CD11c+ cells and NK cells

Single-cell suspensions of pooled lung, lymph node, and murine splenocytes were prepared. CD11c+ cells were isolated by positive selection with magnetic beads (Miltenyi Biotec) conjugated to anti-CD11c; positively selected cells comprised > 96% CD11c^+^ cells, as measured by flow cytometry. NK cells were isolated by negative selection using kits obtained from Miltenyi Biotec. Isolated cells comprised > 97% CD3-NK1.1+ cells, as measured by flow cytometry.

### Flow cytometry and intracellular staining

After the mice were euthanized, the lungs were perfused with 5 ml of PBS via the right ventricle. Lungs were mechanically homogenized and passed through a 70 μm cell strainer. The remaining red blood cells were lysed using BD Pharm Lyse (BD Biosciences). Surface staining to identify leukocyte populations was then performed. For IL-6 intracellular staining, cells (10^6^/ml) were suspended in RPMI 1640 containing 10% FBS and brefeldin A (5 μg/ml), placed in 24-well culture plates, and stimulated with LPS (1 μg/ml), and incubated for a further 4 h at 37°C to allow intracellular accumulation of cytokines. Cells were then permeabilized with 0.1% saponin and stained for intracellular IL-6. The cells were washed, resuspended in FACS buffer, and analyzed by flow cytometry using a FACS Calibur flow cytometer.

### Culture of human blood cells and intracellular cytokine staining

Whole blood was diluted 1:1 with RPMI-1640 medium and cultured in 12-well plates (0.5 × 10^6^ cells/well) in RPMI 1640 containing penicillin/streptomycin (100 U/100 mg/ml), L-glutamine (2 mM), and HEPES (10 mM) (Invitrogen, Carlsbad, CA) in the presence or absence of a PPD (10 μg/ml) at 37°C in a humidified 5% CO_2_ atmosphere. In some cases, an IL-6R neutralizing Ab (2.5 μg/ml) was added to the culture. Brefeldin A (10 μg/ml) was added to the cultures 2 h before the termination of the cultures (total culture time, 6 h). Cells were washed, and red blood cells were lysed with lysis buffer. The cells were fixed using Cytofix/Cytoperm buffer (BD Biosciences) and cryopreserved at -80°C. Intracellular staining for IFN-γ, TNF-α, IL-2, and IL-17A was performed as previously described [9].

### Multiplex ELISA to measure cytokine and chemokine levels in lung homogenates

Mouse multiplex ELISA kits (23-Plex kits, Bio-Rad) were used to measure chemokine and cytokine levels, according to the manufacturer’s instructions.

### Real-time PCR measurement of cytokine mRNA expression

Total RNA was extracted from lung leukocytes or lung tissue as described previously [39]. Total RNA was reverse transcribed using the Clone AMV First-Strand cDNA synthesis kit (Life Technologies). Real-time PCR was performed using the Quantitect SYBR Green PCR kit (Qiagen) in a sealed 96-well microtiter plate (Applied Biosystems) on a spectrofluorometric thermal cycler (7700 PRISM; Applied Biosystems). PCR reactions were performed in triplicate as follows: 95°C for 10 min, followed by 45 cycles of 95°C for 15 s, 60°C for 30 s, and 72°C for 30 s. All samples were normalized to the amount of β-actin/GAPDH transcript present in each sample. The primers used in the study are listed in Table 2.

**Table 2:** List of primers used in the study.

**S. No.**	**Gene Name**	**Mouse Primer Sequences**
**1**	IFN-γ	Forward: TCA AGT GGC ATA GAT GTG GAA GAA Reverse: TGG CTC TGC AGG ATT TTC AT G
**2**	TNF-α	Forward: CAT CTT CTC AAA ATT CGA GTG ACA A Reverse: TGG GAG TAG ACA AGG TAC AAC CC
**3**	IL-17	Forward: CAG ACT ACC TCA ACC GTT CCA C Reverse: TCC AGC TTT CCC TCC GCA TTG A
**4**	IL-6	Forward: TAC CAC TTC ACA AGT CGG AGG C Reverse: CTG CAA GTG CAT CAT CGT TGT TC
**5**	IL-23	Forward: CAT GCT AGC CTG GAA GCG ACA T Reverse: ACT GGC TGT TGT CCT TGA GTC C
**6**	IL-21	Forward: GCC TCC TGA TTA GAC TTC GTC AC Reverse: CAG GCA AAA GCT GCA TGC TCA C
**7**	IL-1β	Forward: CAA CCA ACA AGT GAT ATT CTC CAT G Reverse: GAT CCA CAC TCT CCA GCT
**8**	IL-22	Forward: CAT GCA GGA GGT GGT GCC TT Reverse: CAG ACG CAA GCA TTT CTC AG
**9**	IL-12p35	Forward: CTT AGC CAG TCC CGA AAC CT Reverse: TTG GTC CCG TGT GAT GTC T
**10**	TGF-β	Forward: TGA TAC GCC TGA GTG GCT GTC T Reverse: CAC AAG AGC AGT GAG CGC TGA A
**11**	IL-10	Forward: GGT TGC CAA GCC TTA TCG GA Reverse: ACC TGC TCC ACT GCC TTG CT
**12**	FoXP3	Forward: CCT GGT TGT GAG AAG GTC TTC G Reverse: TGC TCC AGA GAC TGC ACC ACT T
**13**	G-CSF	Forward: TGC TTA AGT CCC TGG AGC AA Reverse: AGC TTG TAG GTG GCA CAC AA
**14**	β-actin	Forward: CTC TGG CTC CTA GCA CCA TGA AGA Reverse: GTA AAA CGC AGC TCA GTA ACA GTC CG

### Histology and IHC

Lungs were inflated and fixed in 10% neutral buffered formalin (v/v) for 24 h. Tissue sections were stained with hematoxylin and eosin. A semi-quantitative analysis was performed using a score from 0 (no inflammation) to 4 (severe inflammation) for each of the following criteria: alveolar wall inflammation, alveoli destruction, leukocyte infiltration, and perivascular inflammation. Immunostaining of thin paraffin-fixed lung sections was performed using antibodies against IL-6 according to the manufacturer’s instructions (Novus Biologicals, USA). Unstained sections of formalin-fixed lung tissue from paraffin blocks were first deparaffinized and subjected to antigen retrieval in a citrate buffer at 95°C, as previously described. Endogenous peroxidase activity was blocked by addition of 3% H_2_O_2_ in methanol. Slides were incubated in 3% BSA in TBS for 2 min, after which primary antibodies were added at predetermined dilutions in TBS-Tween + 1% BSA (1:100) for 1 h at 25°C. Sections were then washed three times in TBS-T for 15 min. The IL-6 antigen was detected by IHC and the DAB (DAKO) chromogen, as previously described. Lung inflammation [40,41] and immunohistochemical readouts were independently assessed by two investigators as previously described [42]. The H-score was determined according to the method described by Pirker et al. Briefly, the percentage of cells with different staining intensities was determined by visual assessment and assigned a score (1+ for light staining, 2+ for intermediate staining, and 3+ for dark staining) using the ImageJ IHC profiler. The H-score was calculated using the formula 1 × (% of 1 + cells) + 2 × (% of 2 + cells) + 3 × (% of 3 + cells).

### Confocal microscopy

Confocal microscopy was performed to colocalize IL-6-producing CD11c+ and NK1.1+ cells in lung sections. Nonspecific binding was blocked with 1% goat serum in PBS for 30 min. The slides were then incubated at 4°C overnight with hamster monoclonal anti-CD11c (Abcam), rabbit polyclonal anti-NK 1.1 (Bioss), and rat monoclonal anti-IL-6 (Novus Bio) antibodies. Subsequently, the slides were washed thoroughly using 1 × PBS. Then, cells were stained with the respective secondary antibodies (goat anti-hamster IgG-Alexa 568, goat anti-rabbit-Alexa 647, or donkey anti-rat-Alexa 488; Life Technologies), washed with PBS, and mounted with Prolong Gold antifade reagent containing DAPI (Life Technologies, USA). The slides were examined and analyzed under a laser-scanning confocal microscope (Zeiss LSM 510 Meta laser-scanning confocal microscope).

### IL-6 bioassay

The efficacy of *in vivo* IL-6 neutralization was assessed in a IL-6 bioassay using the 8G2 cell line (an IL-6-dependent murine hybridoma cell line LS132.8G2) instead of the B9 cell line as previously described [43].

### Statistical analysis

Data analyses were performed using GRAPHPAD PRISM (GraphPad Software, Inc., La Jolla, CA). The results are expressed as the mean ± SE. For normally distributed data, comparisons between groups were performed using a paired or unpaired t-test and ANOVA, as appropriate. Statistically significant differences between two clinical groups were analyzed using the non-parametric Mann–Whitney U-test. Data are deposited in the Dryad Data Repository: (doi:10.5061/dryad.qn42t)[44].

## Supporting Information

S1 Fig Natural killer and dendritic cell interaction enhanced IL-6 production in *Mtb*-infected type 2 diabetic mice. Five control and 5 T2DM mice were infected with 50–100 CFU of aerosolized *Mtb* H37Rv. Six months p.i., lungs from *Mtb*-infected control and T2DM mice were isolated and formalin-fixed. Paraffin-embedded tissue sections were prepared and confocal microscopy analysis was performed to determine NK (pink), IL-6+ (green) and dendritic (red) cell interaction. Scale bar: 20 μm (yellow bar) and 5 μm (white bar).
(TIF)
