# Supplementary figures and images for: NK-CD11c+ Cell Crosstalk in Diabetes Enhances IL-6-Mediated Inflammation during Mycobacterium tuberculosis Infection
Source: PLoS Pathog. 2016 Oct 26;12(10):e1005972. doi: 10.1371/journal.ppat.1005972 (PMC5082658; doi:10.1371/journal.ppat.1005972)

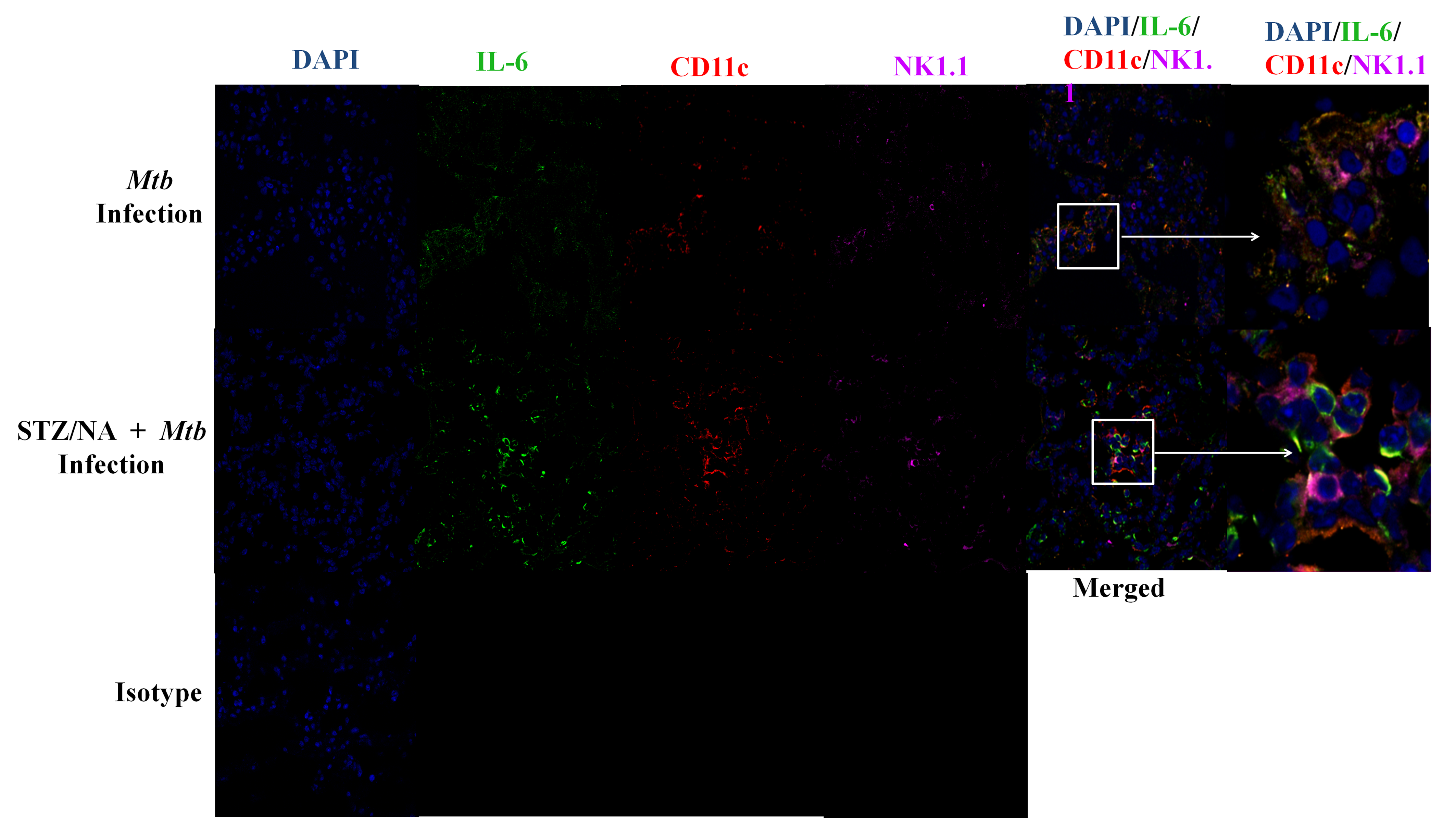

Supplement: S1 Fig — Five control and 5 T2DM mice were infected with 50–100 CFU of aerosolized Mtb H37Rv. Six months p.i., lungs from Mtb-infected control and T2DM mice were isolated and formalin-fixed. Paraffin-embedded tissue sections were prepared and confocal microscopy analysis was performed to determine NK (pink), IL-6+ (green) and dendritic (red) cell interaction. Scale bar: 20 μm (yellow bar) and 5 μm (white bar). (TIF) [file ppat.1005972.s001.tif]
